# The diagnostic value of Xpert detection combined with nanopore sequencing for tuberculosis in HIV/AIDS patients

**DOI:** 10.1016/j.imj.2026.100237

**Published:** 2026-01-13

**Authors:** Chang Song, Chunyan Zhao, Dan Luo, Aichun Huang, Chaoyan Xu, Jieqing Zhong, Yujie Mo, Zhentao Huang, Xiaoshi Lin, Zhouhua Xie, Qingdong Zhu

**Affiliations:** a Department of Tuberculosis, The Fourth People's Hospital of Nanning, Nanning 530023, China; b Clinical medical school, Guangxi Medical University, Nanning 530021, China; c Department of Biostatistics, School of Public Health and Management, Guangxi University of Chinese Medicine, Nanning 530001, China

**Keywords:** Nanopore sequencing, HIV/AIDS, Tuberculosis, Combined diagnosis

## Abstract

**Background:**

Tuberculosis (TB) remains a major cause of death in HIV/AIDS patients, where diagnosis is complicated by atypical presentation and paucibacillary disease. Current diagnostic methods have limitations in speed or sensitivity. This study evaluates the diagnostic value of rapid, portable nanopore sequencing for TB in this high-risk population.

**Methods:**

Clinical data and diagnostic results were collected from 59 HIV/AIDS patients with suspected tuberculosis. The diagnostic performance of nanopore sequencing was compared with AFB staining, TB-DNA testing, Xpert, and solid mycobacterial culture, including sensitivity, specificity, positive predictive value (PPV), negative predictive value (NPV), Kappa coefficient, and the area under the receiver operating characteristic curve (AUC). Additionally, this study further evaluated the diagnostic performance of nanopore sequencing in combination with AFB staining, TB-DNA testing, Xpert, and culture methods.

**Results:**

Nanopore sequencing showed the highest performance among all methods, with a sensitivity of 60.50%, specificity of 90.50%, PPV of 92.00%, NPV of 55.90%, and AUC of 0.76. When used in combination with other methods, nanopore sequencing and Xpert achieved the best diagnostic performance, with a sensitivity of 71.10%, specificity of 85.70%, PPV of 90.00%, NPV of 62.10%, Kappa value of 0.523, and an AUC of 0.78.

**Conclusions:**

The combination of nanopore sequencing and Xpert demonstrates excellent diagnostic performance for diagnosing TB in HIV/AIDS patients.

## Highlights

• Nanopore sequencing enhances TB diagnostic accuracy, achieving 60.50% sensitivity by detecting low pathogen loads and reducing missed cases.
• Combining nanopore sequencing with other methods compensates for individual test limitations, improving overall diagnostic reliability.
• Clinicians should integrate multiple factors, including clinical symptoms and immune status, to interpret nanopore sequencing results effectively.

## 1 Introduction

Tuberculosis (TB), an ancient and highly contagious chronic bacterial disease caused by *Mycobacterium tuberculosis* (MTB), has posed a persistent challenge to global public health. According to the latest WHO statistics for 2024, TB claimed 1.25 million lives in 2023, once again making it the leading cause of death from a single infectious disease worldwide.^1^ Acquired immunodeficiency syndrome (AIDS), caused by the human immunodeficiency virus (HIV), results in severe immunodeficiency by the progressively depleting CD4^+^ T lymphocytes and disrupting their function. This leaves immune system severely compromised, akin to a “barriers full of holes”.^2^^,^^3^ Epidemiological data reveal a grim reality, that is individuals living with AIDS are at a significantly higher risk, dozens of times greater, of contracting MTB compared to the general population. When co-infection occurs, the disease progresses rapidly, with high rates of dissemination and severe forms of TB, resulting in a markedly elevated mortality rate.^4^, ^5^, ^6^ Reports indicate that, among all new TB cases, 662,000 (6.1%) occur in individuals co-infected with HIV/AIDS. Furthermore, autopsy studies have shown that 45.8% of patients who died from TB had not received a clear TB diagnosis during their lifetime.^7^ The clinical manifestations of TB in HIV-infected patients are often subtle and atypical, making diagnosis challenging. Common symptoms such as fever, night sweats, and cough overlap with the immune reconstitution inflammatory syndrome associated with HIV itself, further complicating differential diagnosis. Radiological findings of pulmonary lesions lack specificity, for example, “miliary” patterns can occur in a variety of pulmonary infections. As a result, no single diagnostic method can reliably confirm TB in this population. Additionally, co-management of anti-tuberculosis and antiretroviral therapies poses significant challenges. Drug interactions and overlapping adverse effects, such as hepatotoxicity and myelosuppression can exacerbate clinical outcomes if treatment is not carefully optimized. Furthermore, mycobacterial infections in HIV/AIDS patients accelerate immune deterioration due to a synergistic interplay between pathogens, leading to more rapid disease progression. These factors highlight the critical need for accurate diagnostic strategies to guide timely and effective combination therapy.^8^ Traditional TB diagnostic methods present numerous limitations when applied to HIV/AIDS patients. Acid-fast bacilli (AFB) staining microscopy, while simple and cost-effective, suffers from poor sensitivity, as it requires a substantial bacterial load to detect infection, frequently missing early or paucibacillary cases.^9^ Mycobacterial culture, considered the “gold standard”, is another commonly used method, but it is time-consuming, often requiring several weeks for results. This delay is incompatible with the urgent clinical need for rapid diagnosis and treatment. Moreover, the immunocompromised state of HIV/AIDS patients increases the risk of sample contamination and overgrowth other microorganisms, further compromising diagnostic accuracy. Additionally, mycobacterial culture requires specialized infrastructure, including Biosafety Level 3 laboratories, and rapid sample transport of these facilities, limiting its applicability in resource-constrained settings.^10^

There is an urgent need for an accurate, rapid, and cost-effective diagnostic method to enable early and precise diagnosis of TB in patients with HIV/AIDS. Nanopore sequencing technology represents a revolutionary advancement in next-generation nucleic acid sequencing. This technique uses nanoscale pore structures and the electrical properties of nucleic acid molecules for sequencing.^11^ Its core principle involves the passage of single-stranded nucleic acid molecules (DNA or RNA) through nanoscale pore channels embedded in a biological membrane or solid-state material, driven by an electric field. Due to the differences in chemical structure and spatial conformation of the nucleotide bases, these molecules cause specific changes in the ion current intensity and blockage duration within the channel. Highly sensitive current detection devices capture and interpret these electrical signals, which are subsequently converted into base sequence information through complex algorithms.^12^, ^13^, ^14^ Compared to traditional sequencing technologies, nanopore sequencing offers several notable advantages. It has relatively relaxed requirements for the purity and integrity of nucleic acids, allowing direct analysis of crudely extracted samples.^15^ This is particularly advantageous for HIV-infected patients, whose weakened immune systems often lead to variability in the quality of collected specimens. Additionally, the process is straightforward and rapid, eliminating the need for labor-intensive polymerase chain reaction (PCR) amplification cycles, which are prone to bias in traditional second-generation sequencing. This significantly shortens the diagnostic timeline, and allowing preliminary results to be obtained within hours, a critical advantage in addressing the urgent need for TB diagnostic in HIV/AIDS patients. Moreover, nanopore sequencing produces exceptionally long sequencing reads, facilitating the acquisition of complete gene segments and enabling the analysis of complex genomic structural variations. This is particularly beneficial for the identification for pathogens like MTB, whose genomes are rich in repetitive sequences and structural polymorphisms. Despite its potential, few studies have systematically assessed the application of nanopore sequencing technology in TB diagnosis within this immunocompromised patient population, such as those with HIV/AIDS.

In light of these gaps, this study is the first time to compare nanopore sequencing with other diagnostic methods, including smear AFB staining, TB-DNA testing, Xpert, and solid culture of mycobacteria, to comprehensively evaluate the diagnostic efficacy of nanopore sequencing for TB patients with HIV/AIDS.

## 2 Materials and methods

### 2.1 Subject sample inclusion

This study included patients suspected of having HIV/AIDS complicated with TB who visited the Fourth People's Hospital of Nanning between October 2021 to August 2024. Diagnostic methods utilized included AFB staining, TB-DNA testing, mycobacterial solid culture, and nanopore sequencing. The requirement for informed consent to study inclusion and the need for consent to participate were waived by the Ethics Committee of The Fourth People's Hospital of Nanning because of the lack of study intervention in patient diagnosis and treatment and the retrospective nature of the study. This study is in accordance with the Declaration of Helsinki. Ethical approval was granted by the hospital's ethics committee (Ethical Approval Number: [2023]24). Inclusion criteria were as follows: (1) Confirmed infection with the HIV or diagnosed with AIDS, verified through enzyme-linked immunosorbent assay screening and Western blotting or immunofluorescence testing, with an official diagnostic certificate. (2) Presentation with symptoms suggestive of TB, such as cough lasting more than 2 weeks (with or without sputum), persistent or intermittent fever over 38°C, night sweats disturbing sleep, weight loss exceeding 5% of original body weight within 3 months, and miliary nodules on pulmonary computed tomography. (3) Availability of complete demographic and clinical information and willingness to cooperate with diagnostic procedures until confirmation or exclusion of TB. Exclusion criteria included: (1) Diagnosed non-tuberculous other pulmonary diseases such as lung cancer, pneumonia, lung abscess, or bronchiectasis without the possibility of co-occurring TB. (2) History of anti-tuberculosis treatment within the past 3 months. (3) Severe comorbid conditions, including advanced liver and kidney failure, acute cardiovascular and cerebrovascular diseases, active phase of malignant hematological diseases, and other severe diseases. (4) Severe mental illnesses or cognitive disorders, such as schizophrenia, severe depression that prevent cooperation. (5) Pregnancy or lactation (patients could be reconsidered after completing these phases if they met all other inclusion criteria).

### 2.2 Diagnostic criteria

Subjects were diagnosed with AIDS in accordance with the criteria outlined in the Chinese AIDS Diagnosis and Treatment Guidelines (2021 Edition).^16^ The diagnosis of TB was based on the Pulmonary Tuberculosis Diagnostic Criteria (WS 288-2017)^17^ proposed by the National Health and Family Planning Commission of the People's Republic of China. The present study is based mainly on etiological examination results (including bacteriology and molecular biology), and combined with epidemiological history, clinical manifestations, chest imaging findings, relevant ancillary examinations, and differential diagnosis for the comprehensive clinical diagnosis. However, a diagnosis of pulmonary tuberculosis can be confirmed or clinically made if one or more of the following criteria are met: (1) Positive culture or molecular biology test for MTB: A definitive diagnosis was made if MTB was detected through culture or molecular biology testing methods. (2) Pulmonary tissue pathology consistent with pulmonary tuberculosis: Pulmonary tissue pathology demonstrating typical TB-related pathological features, such as caseous necrosis or epithelioid granulomas, confirmed the diagnosis. (3) Response to anti-tuberculosis treatment: For sputum smear-negative patients, a clinical diagnosis of pulmonary tuberculosis was considered. Preventive anti-tuberculosis treatment was initiated for three months, and a reduction or resolution of pulmonary lesions during this period confirmed the diagnosis.

### 2.3 Sample collection for detection

This study employed fresh sputum and bronchoalveolar lavage fluid (BALF) specimens for related testing. The collection of sputum specimens required patients to rinse their mouths with water for at least 1 minute per rinse, repeated three times. After performing a deep inhalation followed by a forceful exhalation, patients were instructed to cough up sputum from the lower respiratory tract. The sputum was then collected into a sterile container, ensuring a sample volume between 3 and 5 mL. Specimens were stored at a temperature of 2 to 8°C to maintain their integrity. The collection of BALF specimens involved the following procedure: First, topical anesthetics were applied to lubricate the patient's nostrils and the bronchoscope. The bronchoscope was then inserted through the nasal or oral cavity. For patients with tracheal intubation or tracheostomy, the insertion was performed through the artificial airway. The bronchoscope's tip was placed near the opening of the target bronchial segment or subsegment. Through the operating channel, a total of 60 to 100 mL of sterilized saline, pre-warmed to 37°C, was instilled in 5 aliquots, each comprising 20 to 50 mL. The lavage fluid was recovered using negative pressure suction at levels below −100 mmHg. The BALF sample was finally aspirated into a sterile container. The collected specimens were used for various diagnostic and research purposes, including phenotypic drug susceptibility testing, Xpert, fluorescent PCR melting curve analysis, and nanopore sequencing detection. Additionally, for patients requiring further diagnostic precision, puncture procedures guided by B-mode ultrasound were conducted to obtain fluid or tissue samples from the thoracic cavity, abdomen, or other lesion sites.

### 2.4 AFB staining

Patient-derived samples, including sputum, BALF, puncture fluid were used for AFB staining. Smears were prepared on microscope slides following the Ziehl-Neelsen staining smear preparation protocol.^18^ In this study, fluorescent auramine O staining method was employed. The set included auramine O solution (containing auramine O and phenol) (Cat number, Zhuhai Beso Biotechnolog, Zhuhai, China), acid-alcohol solution (containing ethanol and hydrochloric acid), and 5% potassium permanganate solution. The prepared slides were fixed by flame. Auramine O staining solution was added to completely cover the slide, and incubated for 30 min. Then, the slides were gently rinsed with running water from one end to remove the staining solution and excess moisture was drained. A decolorizer was applied along the upper edge of the slide to cover the smear completely. The slides were decolorized for 3 min (or until the color disappeared) and then gently rinsed with running water to remove the decolorizer. A counterstain was added for 1 minute, followed by gently rinsed again with running water and air drying. The slides were examined under a fluorescent microscope, where acid-fast bacteria appeared as yellow, rod-shaped and slightly curved structures against a dark background.

### 2.5 TB-DNA testing

The nucleic acid detection kit was used in this study combines dual PCR technology and Taqman probe technologies for the qualitative detection of mycobacterial DNA extracted from clinical samples (Bio-Rad Laboratories, California, America). The assay was designed with specific sequences to differentiate between the MTB complex and non-tuberculous mycobacteria. Primers and probes were labeled with distinct fluorescent markers, allowing the detection of fluorescence signal changes through different fluorescence channels, which is possible to identify the MTB complex and non-tuberculous mycobacteria. Real-time fluorescent quantitative PCR was performed using the Applied American Applied Biosystems 7500 (Shanghai Hongshi SLAN-96S, and SLAN-96P, Shanghai, China). The reagents, provided by Chengdu Bio-chip Science & Technology Co., Ltd., Chengdu, China, were specifically designed for mycobacterial DNA detection using the PCR-fluorescence probe method.

### 2.6 Xpert

The Xpert was conducted using a dedicated pipette to aspirate 2 mL of liquefied sample into the test cartridge. Care was taken to perform this operation slowly to minimize the risk of aerosol formation. Once the sample was added, the test was initiated within 30 min to ensure sample integrity. Before testing, it was confirmed that the GeneXpert Dx software was installed and configured appropriately for the Xpert assay. The operating steps for the Xpert test included the following: (1) Powering on the instrument. (2) Logging into the GeneXpert system. (3) Creating a new test in the software. (4) Scanning the patient and sample barcodes. (5) Scanning the barcode on the test cartridge. (6) Starting the test and placing the test cartridge into the instrument module. Upon completion of the test, the test cartridge was removed and disposed of following standard biosafety protocols. Routine maintenance of the GeneXpert instrument was performed to ensure optimal performance. This included cleaning the instrument's surface and test chamber with alcohol wipes, and replacing or cleaning the air filter at the fan annually.

### 2.7 Solid mycobacterial culture

The procedure for solid mycobacterial culture involved preparing a 4% NaOH solution by dissolving 4 g of NaOH in 100 mL of distilled, followed by high-pressure sterilization. A 5 mL sample was mixed with an equal volume of 4% NaOH solution, vortexed for 20 seconds, and incubated at room temperature for 15 min. After that, pH 7.2 phosphate buffer solution was added, and the mixture underwent centrifugal sedimentation. The entire processing procedure was completed within 20 min after the addition of the NaOH solution to ensure sample viability. The treated sample (0.1 mL) was inoculated onto a modified Loewenstein culture medium (Zhuhai Beso Technology Co., Ltd., Zhuhai, China) under sterile conditions. After inoculation, the cultures were incubated and observed for growth on days 3 and 7, and then monitored weekly. Positive results were immediately confirmed by smear microscopy and reported. A positive culture within 7 days indicated the presence of rapid-growing mycobacteria, whereas growth observed after 7 days was categorized as slow-growing mycobacteria. Cultures with no visible growth after 8 weeks were reported as negative.

### 2.8 Nanopore sequencing

This study employed the third-generation nanopore sequencing workflow provided by Hangzhou Saint Medical Technology Co., Ltd., Hangzhou China, which includes multiple steps, such as pre-treatment, sample preparation, nucleic acid extraction, nucleic acid quality inspection, PCR, nanopore sequencing library construction, and nanopore sequencing and data analysis. During the pre-treatment phase, laboratory personnel disinfected the biological safety cabinet and experimental workbench in the reagent preparation area, and prepared the required reagents and samples. The sample preparation phase involved the pre-treatment and grinding of samples, ensuring that all operations were conducted within a biological safety cabinet to maintain biosafety standards. Nucleic acid extraction was performed using a magnetic bead-based purification method after the preparation of extraction reagents and processing of the samples. The extracted nucleic acids were then subjected to quality inspection using the Qubit 4.0 fluorometer to assess their concentration and integrity (Thermo Fisher Scientific, Massachusetts, America). The PCR reaction system was assembled using a 2 × PCR mix, primers, DNA template, and ddH_2_O. Amplification was carried out using a thermal cycler, and the resulting PCR products were purified with magnetic beads. Following purification, barcode labeling and Qubit quantification were performed to ensure the quality of the PCR products before proceeding to library construction. The prepared library was then loaded onto the GridION nanopore sequencing platform, with real-time sequencing data captured and analyzed using MinKNOW software. The data analysis phase consisted of several critical steps, including quality filtering of sequencing reads, removal of host DNA sequences, and alignment of the remaining reads against pathogen reference databases and antimicrobial resistance gene databases. All procedures were strictly conducted in accordance with the standard operating procedures to ensure accuracy and reliability of sequencing results.

### 2.9 Statistical analysis

All analyses were performed with SPSS 23.0 (IBM Corp., Chicago, IL, USA). Qualitative data are expressed as rates or percentages. After testing normality with the Kolmogorov–Smirnov test, quantitative data with normal distribution are presented as mean ± SD. Categorical variables are reported as *n* (%) and compared by *χ*² test or Fisher's exact test when expected frequencies were < 5. Using the clinician-made tuberculosis diagnosis as the reference standard, 2 × 2 contingency tables were constructed to calculate the diagnostic performance metrics of nanopore sequencing: sensitivity, specificity, positive predictive value (PPV), negative predictive value (NPV), Kappa coefficient, and area under the receiver operating characteristic curve (AUC). *p* < 0.05 was considered statistically significant.

## 3 Results

### 3.1 Patient clinical baseline information

A total of 59 patients were included in this study, comprising 38 non-TB patients and 21 TB patients. The baseline characteristics of the study population are shown in Table 1. In terms of age distribution, the average age of the total population was 50.15 years. The mean age of non-TB patients was 51.76 years, while that of TB patients was 49.26 years. Regarding sex distribution, the total cohort included 46 men (77.97%) and 13 women (22.03%). Among the non-TB patients, there were 18 men (85.71%) and 3 women (14.29%). For the TB patients, 28 men (73.68%) and 10 women (26.32%) were identified. (*p* > 0.05)

**Table 1:** Patient clinical baseline.

Items	Total	TB	Non-TB	*T/χ*²	*p*
Number	59	38	21	—	—
Age (years)	50.15 ± 13.37	49.26 ± 13.81	51.76 ± 12.71	−0.684	0.497
Sex					
Male	46	28	18	1.139	0.286
Female	13	10	3	—	—
Immune cell counts (cells/μL)					
CD3^+^	793.17 ± 572.34	772.89 ± 592.55	829.86 ± 546.07	−0.363	0.718
CD4^+^	219.78 ± 282.09	227.13 ± 309.43	206.48 ± 230.97	0.267	0.790
CD8^+^	546.68 ± 394.06	512.03 ± 369.80	609.38 ± 436.95	−0.907	0.368

*Abbreviation*: TB, tuberculosis.

Immune cell counts were also evaluated. The mean CD3^+^ T cells count for the total cohort was 793.17 cells/µL, with averages of 829.86 cells/µL and 772.89 cells/µL for the non-TB and TB patient group, respectively. The mean CD4^+^ T cell count was 219.78 cells/µL for the total cohort, with averages of 206.48 cells/µL in the non-TB patient group and 227.13 cells/µL in the TB patient group. Similarly, the average CD8^+^T cell count was 546.68 cells/µL for the total cohort, with values of 609.38 cells/µL in the non-TB patient group, and 512.03 cells/µL in the TB patient group. (*p* > 0.05)

In our study, bronchoalveolar lavage fluid was the most common type of sample, accounting for 44.07% of the total number of samples, followed by cerebrospinal fluid (16.95%), sputum (8.47%), and peripheral blood (8.47%), etc. (Table 2).

**Table 2:** Types and proportions of specimens collected for analysis.

Sample type	Number	Rate (%)
Bronchoalveolar lavage fluid	26	44.07
Cerebrospinal fluid	10	16.95
Sputum	5	8.47
Peripheral blood	5	8.47
Puncture tissue	4	6.78
Pleural effusion	4	6.78
Abscess aspiration	3	5.08
Bone marrow	2	3.39

### 3.2 Detection results of different detection methods

**Fig. 1:**
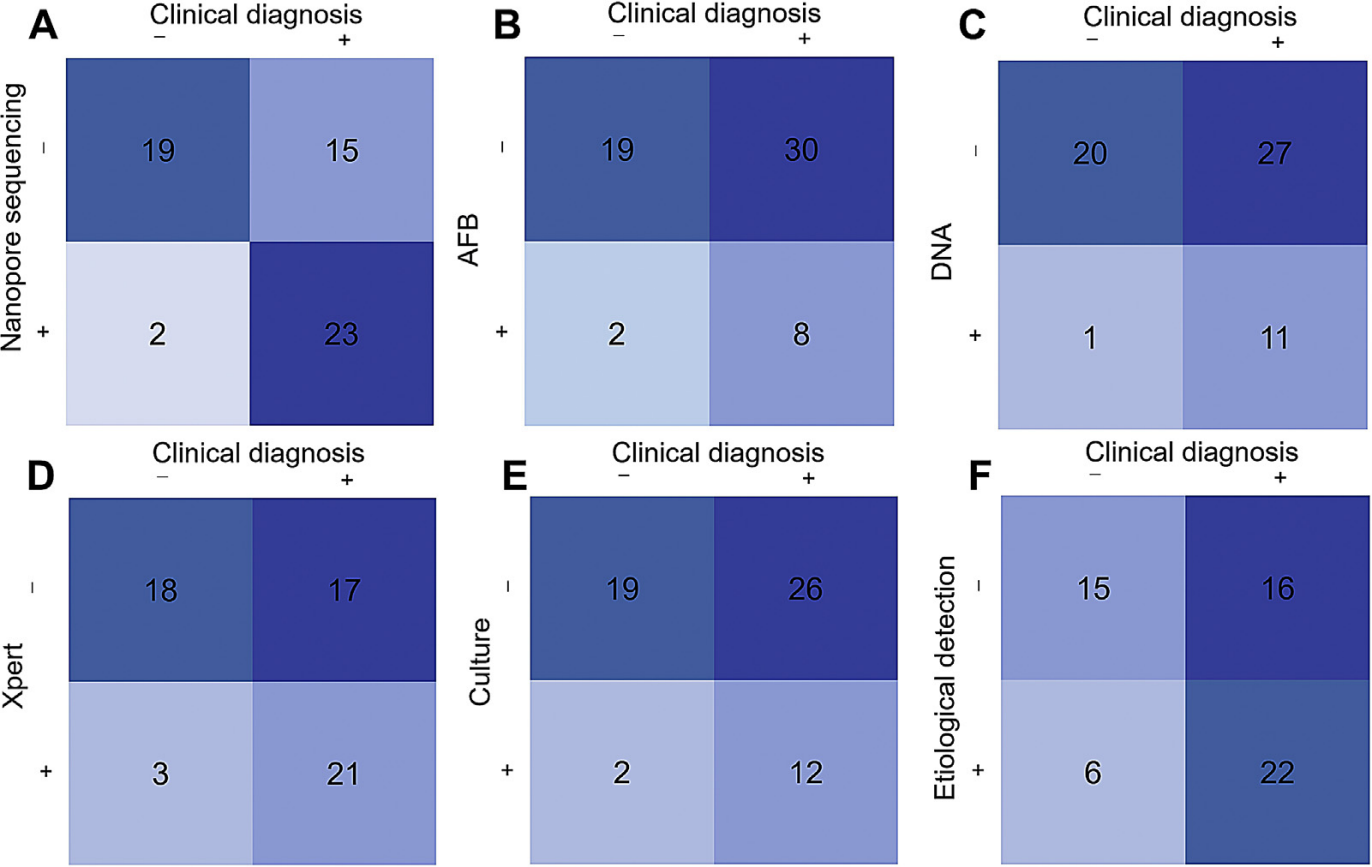
Diagnostic four-fold table of various detection methods. *Abbreviation*: AFB, acid-fast bacilli.

The comparison of detection results between different methods and clinical diagnosis is illustrated in Fig. 1. Nanopore sequencing demonstrated the highest accuracy, correctly identifying 23 cases of TB. In contrast, the four conventional detection methods, AFB staining, TB-DNA testing, Xpert, and mycobacterial solid culture, missed a substantial number of positive cases, reporting 30, 27, 17, and 26 false negatives, respectively. When traditional detection methods were combined into a “microbiological detection” category, the combined approach identified 22 positive cases, which was still inferior to the performance of nanopore sequencing. This highlights the enhanced diagnostic capability of nanopore sequencing in identifying tuberculosis cases, as it outperformed conventional methods in terms of accuracy and detection rate.

### 3.3 Comparison of diagnostic efficacy between nanopore sequencing and conventional detection methods

As shown in Table 3, nanopore sequencing demonstrated superior diagnostic performance compared to conventional detection methods significantly. It achieved a sensitivity of 60.50%, specificity of 90.50%, PPV of 92.00%, NPV of 55.90%, and a Kappa coefficient of 0.428. In contrast, TB-DNA testing showed high specificity (95.20%) but significantly lower sensitivity (28.90%), indicating its limited ability to detect TB cases. Both Xpert and culture methods exhibited moderate sensitivity and specificity. Xpert achieved a sensitivity of 55.30% and specificity of 85.70%, while the culture method had a sensitivity of 31.60% and specificity of 90.50%. AFB staining had a specificity comparable to nanopore sequencing (90.50%), but its sensitivity was the lowest among the methods tested, at only 21.10%. When the results of the traditional detection methods (including AFB, TB-DNA, Xpert, and Culture) were combined and categorized as “Etiological testing,” the overall sensitivity increased to 57.90% and specificity to 71.40%. However, these values remained lower than those of nanopore sequencing. Diagnostic performance was further evaluated by comparing the AUC. A higher AUC value indicates better diagnostic efficacy. Nanopore sequencing achieved the highest AUC value of 0.76 with a confidence interval of 0.63–0.88, outperforming AFB staining (AUC = 0.56), TB-DNA testing (AUC = 0.62), Xpert (AUC = 0.71), and mycobacterial solid culture (AUC = 0.61).

**Table 3:** Diagnostic performance of nanopore sequencing, AFB, TB-DNA detection, Xpert, and mycobacterial solid culture.

Method	Sensitivity (%)	Specificity (%)	PPV (%)	NPV (%)	Kappa	*χ*²	*p*	AUC
Nanopore sequencing	60.5	90.5	92.0	55.9	0.448	14.409	< 0.001	0.76 (95% CI: 0.63–0.88)
AFB	21.1	90.5	80.0	38.8	0.089	1.277	0.258	0.56 (95% CI: 0.41–0.71)
TB-DNA	28.9	95.2	91.7	42.6	0.189	4.883	0.027	0.62 (95% CI: 0.48–0.76)
Xpert	55.3	85.7	87.5	51.4	0.357	9.412	0.002	0.71 (95% CI: 0.57–0.84)
Culture	31.6	90.5	85.7	42.2	0.176	3.635	0.057	0.61 (95% CI: 0.47–0.76)
Etiological testing	57.9	71.4	78.6	48.4	0.265	4.664	0.031	0.65 (95% CI: 0.50–0.79)

*Abbreviations*: PPV, positive predictive value; NPV, negative predictive value; AUC, area under the receiver operating characteristic curve; TB, tuberculosis; AFB, acid-fast bacilli.

### 3.4 Detection outcomes of combined diagnostics with nanopore sequencing

**Fig. 2:**
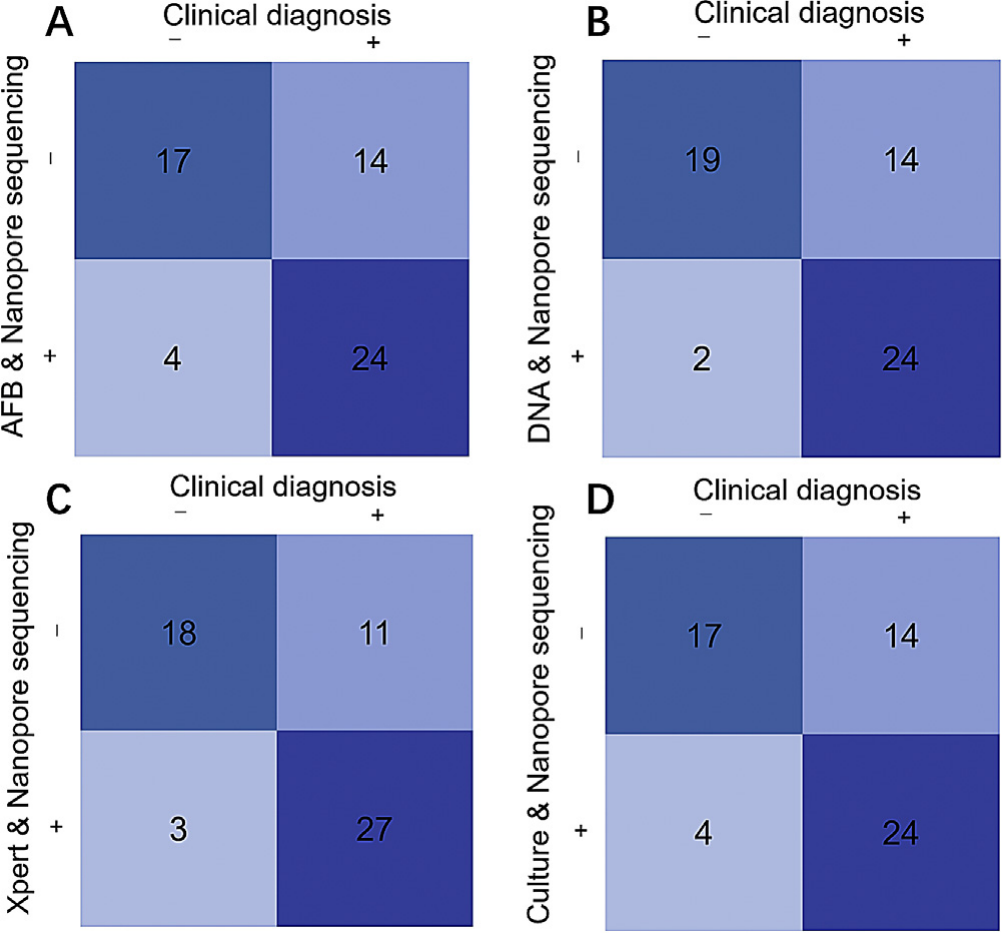
Diagnostic four-fold table of combined detection methods.

Although nanopore sequencing demonstrated significantly advantages over conventional detection methods, its use alone still resulted in certain missed cases. Missed diagnoses could potentially delay treatment, and contribute to disease progression. To address this, the diagnostic performance of combining nanopore sequencing with other methods was further analyzed, such as AFB staining, TB-DNA testing, Xpert, and mycobacterial structure culture methods. As shown in Fig. 2, the comparison results of different combined detection methods with clinical diagnosis showed that AFB staining & nanopore sequencing, TB-DNA testing & nanopore sequencing, Xpert & nanopore sequencing, and mycobacterial structure culture & nanopore sequencing correctly identified 24, 24, 27, and 24 positive cases, respectively, and correctly excluded 17, 19, 18, and 17 negative cases, respectively. These findings suggest that combining nanopore sequencing with other methods improves diagnostic accuracy by compensating for the limitations of individual tests.

### 3.5 Diagnostic efficacy comparison of combined diagnostics with nanopore sequencing

The diagnostic efficacy of the combined methods was further evaluated, and the results are summarized in Table 4. Among all the combined detection methods, Xpert & nanopore sequencing demonstrated the highest diagnostic performance. It exhibited superior sensitivity, specificity, and predictive values, along with the highest AUC value (0.78), significantly improving the diagnostic accuracy for TB. This indicates that combining Xpert with nanopore sequencing offers the most reliable approach for clinical diagnosis.

**Table 4:** Diagnostic efficacy comparison of nanopore sequencing combined with conventional detection methods.

Method	Sensitivity (%)	Specificity (%)	PPV (%)	NPV (%)	Kappa	χ²	*p*	AUC
AFB & Nanopore sequencing	63.2	81.0	85.7	54.8	0.399	10.554	0.001	0.72 (95% CI: 0.59–0.86)
TB-DNA & Nanopore sequencing	63.2	90.5	92.3	57.6	0.476	15.785	< 0.001	0.77 (95% CI: 0.65–0.89)
Xpert & Nanopore sequencing	71.1	85.7	90.0	62.1	0.523	17.439	< 0.001	0.78 (95% CI: 0.66–0.91)
Culture & Nanopore sequencing	63.2	81.0	85.7	54.8	0.399	10.554	0.001	0.72 (95% CI: 0.59–0.86)

*Abbreviations*: PPV, positive predictive value; NPV, negative predictive value; AUC, area under the receiver operating characteristic curve; TB, tuberculosis; AFB, acid-fast bacilli.

## 4 Discussion

Nanopore sequencing technology, as an emerging molecular diagnostic tool, has shown great potential in the diagnosis of TB.^19^, ^20^, ^21^ This technology uses protein nanopores as biosensors to detect individual DNA or RNA molecules passing through the nanopore, enabling real-time sequencing of fragments up to tens of thousands of base pairs in length.^11^ Compared to traditional second-generation sequencing technologies, third-generation nanopore sequencing not only achieves taxonomic resolution that short-read sequencing platforms (< 300) cannot, but also significantly reduces sample preparation time, thereby accelerating the overall detection process.^22^ Advances in integrated circuits and computer hardware have driven the miniaturization of nanopore sequencing devices. This evolution has introduced a new phase in the technology, with devices becoming increasingly portable and even handheld. This transformation enables nanopore sequencing to leverage computing resources from laptops, tablets, and other portable mobile devices, greatly expanding its application scenarios and improving convenience.^23^ For TB patients with HIV, nanopore sequencing is particularly advantageous due to its rapid, high-throughput, and absence of labor-intensive library construction steps. In low-resource settings, its portable equipment and ease of operation allow on-site diagnostics, significantly enhancing the ability to quickly and accurately detect MTB.

To effectively prevent dual infections of HIV and TB and reduce associated mortality, it is crucial to accurately identify individuals at risk of co-infection, and promote timely anti-tuberculosis treatment.^24^ Despite its long-standing use as a conventional diagnostic method for detecting TB in many countries, traditional sputum smear microscopy has remained largely unchanged for over a century and suffers from significant limitations in sensitivity.^25^ The diagnostic mechanism relies on the high lipid content in the MTB cell wall, which provides acid resistance. When stained with specific dyes, the bacterial cell wall resists acid-alcohol decolorization, a property that confers acid-fastness to the organism.^26^ While this method is widely used for its affordability and speed, it has low and unstable sensitivity, ranging between 20% and 60%, especially in cases of HIV-associated TB.^27^^,^^28^ This limitation is consistent with the findings of this study, where the sensitivity of AFB staining was 23.10%, highlighting its inability to detect many early or mild infections. Mycobacterial culture is considered the gold standard for TB diagnosis, because it directly confirms infection by culturing MTB on specific media. However, despite its high specificity, the sensitivity of mycobacterial culture often falls short of clinical diagnostic requirements in practice.^29^^,^^30^ In this study, the sensitivity of mycobacterial culture was 35.90%. Another significant limitation of mycobacterial culture is the prolonged detection time, typically requiring 2–8 weeks to obtain results. This delay poses a significant challenge for TB patients who require early diagnosis and treatment.^31^^,^^32^ Additionally, the method requires specialized laboratory equipment and strict operating conditions, which are often unavailable in primary healthcare facilities, further limiting its accessibility. Molecular diagnostic techniques have made significant breakthroughs in the field of TB detection. For example, the Xpert recommended by the WHO, employs nucleic acid amplification technology.^33^ However, Xpert has limitations in detecting low-concentration bacterial samples and cannot distinguish whether TB is in an active state. Furthermore, its high cost and complex sample processing steps pose barriers to its widespread clinical application, particularly in resource-limited settings.

Through the evaluation of various detection methods, it was observed that nanopore sequencing exhibits unique advantages in multiple diagnostic performance indicators. Its sensitivity of 60.50% represents a significant improvement compared to other traditional detection methods, enabling it to more effectively identify true TB cases and reduce missed diagnoses. The higher sensitivity is attributed to the ability of nanopore sequencing to comprehensively detect the entire genome or specific gene regions of MTB, facilitating detection even at low pathogen loads.

However, nanopore sequencing has its own drawbacks when used independently. Missed cases indicate challenges, such as potential inefficiencies in sample processing or sequencing depth. To address challenges, combined diagnostic strategies are of great importance. The results showed that different combinations of Xpert and nanopore sequencing demonstrated the best diagnostic performance. Xpert serves as an initial screening tool, quickly excluding non-TB cases and reducing the workload and cost of subsequent diagnostic steps. For samples identified as positive or suspected positive by Xpert, nanopore sequencing provides deeper genomic analysis of MTB, thereby improving diagnostic accuracy, especially for complex or mixed-infection cases. Additionally, the separate comparison results showed that Xpert had the highest sensitivity in centralized routine tests. Combined with the excellent high specificity of nanopore sequencing, the two together can more easily and accurately detect TB. In comparison, other combined strategies also improved diagnostic efficiency, but to a lesser extent. AFB staining relies on morphological observation of AFB under a microscope, which differs fundamentally from the genomic analysis offered by nanopore sequencing, limiting complementary effect. While both TB-DNA testing and nanopore sequencing are based on nucleic acid detection, differences in gene targets, sensitivity, and specificity reduce the complementary benefits when combined. Mycobacterial culture methods have long culture period and susceptibility to false negatives make the combination less effective in improving speed and accuracy than Xpert combined with nanopore sequencing.

Nanopore sequencing, as a cutting-edge molecular diagnostic tool, should be incorporated into the diagnostic workflow TB, especially in complex cases where traditional methods fail or when results from different detection methods are contradictory. For institutions with the technical conditions and resources to implement nanopore sequencing, combining it with Xpert is suggested as the optimal strategy for in diagnosing TB. In cases of suspected TB that are difficult to diagnose using conventional methods, comprehensive consideration of multiple factors is critical, including the clinical manifestations of the patient, medical history, and epidemiological data. For patients with impaired immune function, even a negative nanopore sequencing result may not entirely rule out TB. Immune dysfunction can lead to low bacterial load or altered gene expression, which may reduce the accuracy of sequencing tests.^34^^,^^35^ In such cases, a broader diagnostic approach is required, integrating clinical assessment, imaging studies, auxiliary tests, and other diagnostic tools to improve diagnostic precision.

Unlike previous studies that focused solely on patients with TB,^36^ this study zeroes in on the special group of individuals co-infected with HIV and TB. Owing to the compromised immune function caused by HIV infection, the complexity of the condition and the difficulty of diagnosis are significantly heightened in this group. Nevertheless, even under such complex circumstances, nanopore sequencing technology still demonstrates outstanding detection performance, being able to accurately and swiftly identify the presence of MTB. Moreover, this study innovatively integrates nanopore sequencing with other traditional pathogen detection methods for combined diagnosis. This combined diagnostic strategy not only leverages the strengths of multiple detection techniques to enhance diagnostic accuracy and comprehensiveness but also provides clinicians with richer information, facilitating more precise treatment planning.

While this study highlights the potential of nanopore sequencing in TB diagnostics, it also has certain limitations. The small sample size may introduce bias, and the regional restriction of the sample population may limit the generalizability of the findings. Future research should aim to include larger sample sizes and conduct multi-regional, multicenter studies to validate and expand on these findings. Additionally, further investigation into the role of nanopore sequencing in diagnosing TB among HIV/AIDS populations is warranted, given the specific challenges associated with co-infection.

## 5 Conclusion

The combination of nanopore sequencing and Xpert demonstrates excellent diagnostic performance for diagnosing TB in HIV/AIDS patients. This approach has the potential to serve as an effective diagnostic method, significantly improving TB detection rates in this population.

## CRediT authorship contribution statement

**Chang Song:** Software, Methodology, Formal analysis, Data curation, Investigation. **Chunyan Zhao:** Writing – original draft, Software, Resources, Data curation. **Dan Luo:** Visualization, Software, Investigation, Data curation, Formal analysis. **Aichun Huang:** Writing – review & editing, Methodology, Funding acquisition, Conceptualization. **Chaoyan Xu:** Writing – review & editing, Methodology, Conceptualization. **Jieqing Zhong:** Writing – review & editing, Methodology, Conceptualization. **Yujie Mo:** Writing – review & editing, Methodology, Conceptualization. **Zhentao Huang:** Writing – review & editing, Methodology, Conceptualization. **Xiaoshi Lin:** Writing – review & editing, Methodology, Conceptualization. **Zhouhua Xie:** Writing – review & editing, Supervision, Project administration, Data curation, Writing – original draft. **Qingdong Zhu:** Writing – review & editing, Supervision, Project administration, Funding acquisition, Funding acquisition, Writing – original draft.

## Informed consent

The requirement for informed consent to study inclusion and the need for consent to participate were waived by the Ethics Committee of The Fourth People's Hospital of Nanning because of the lack of study intervention in patient diagnosis and treatment and the retrospective nature of the study.

## Organ donation

Not applicable.

## Ethics statement

The study protocol was approved by the Ethics Committee of The Fourth People's Hospital of Nanning (Ethical Approval Number: [2023]24). This study is in accordance with the Declaration of Helsinki.

## Data availability statement

The datasets analyzed during the current study are not publicly available but are available from the corresponding author on reasonable request.

## Animal treatment

Not applicable.

## Generative AI

Generative AI was not used in the writing and revision of this manuscript.

## Funding

This research was funded by the Guangxi Zhuang Autonomous Region Health Commission Self-financed Scientific Research Project, grant numbers Z-A20231211 and Z20210352.

## Declaration of competing interest

The authors declare that they have no known competing financial interests or personal relationships that could have appeared to influence the work reported in this paper.
